# Preventing dispensing errors by alerting for drug confusions in the pharmacy information system—A survey of users

**DOI:** 10.1371/journal.pone.0197469

**Published:** 2018-05-29

**Authors:** Zizi Campmans, Arianne van Rhijn, René M. Dull, Jacqueline Santen-Reestman, Katja Taxis, Sander D. Borgsteede

**Affiliations:** 1 Department of Clinical Decision Support, Health Base Foundation, Houten, the Netherlands; 2 Department of PharmacoTherapy, -Epidemiology & -Economics (PTEE), University of Groningen, Groningen, the Netherlands; 3 Portal for patient safety/Central Medication incidents Registration, Utrecht, the Netherlands; 4 SAL pharmacy Schuytgraaf, Arnhem, the Netherlands; 5 Department of Hospital Pharmacy, Erasmus University Medical Center, Rotterdam, the Netherlands; Universita degli Studi di Napoli Federico II, ITALY

## Abstract

**Introduction:**

Drug confusion is thought to be the most common type of dispensing error. Several strategies can be implemented to reduce the risk of medication errors. One of these are alerts in the pharmacy information system.

**Objective:**

To evaluate the experiences of pharmacists and pharmacy technicians with alerts for drug name and strength confusion.

**Methods:**

In May 2017, a cross-sectional survey of pharmacists and pharmacy technicians was performed in community pharmacies in the Netherlands using an online questionnaire.

**Results:**

Of the 269 respondents, 86% (n = 230) had noticed the alert for drug name confusion, and 26% (n = 67) for drug strength confusion. Of those 230, 9% (n = 20) had experienced that the alert had prevented dispensing the wrong drug. For drug strength confusion, this proportion was 12% (n = 8). Respondents preferred to have an alert for drug name and strength confusion in the pharmacy information system. ‘Alert fatigue’ was an important issue, so alerts should only be introduced for frequent confusions or confusions with serious consequences.

**Conclusion:**

Pharmacists and pharmacy technicians were positive about having alerts for drug confusions in their pharmacy information system and experienced that alerts contributed to the prevention of dispensing errors. To prevent alert fatigue, it was considered important not to include all possible confusions as a new alert: the potential contribution to the prevention of drug confusion should be weighed against the risk of alert fatigue.

## Introduction

Dispensing errors occur in clinical and community pharmacy and commonly involve supply of the wrong drug, the wrong strength and the wrong form of medication [1,2]. They occur at an average rate of 4 in 250 prescriptions (1.6%) in pharmacies in the United States [3], and incidences up to 45% have been reported in different pharmacy settings [4]. A study in the Netherlands showed that 41% of all medication incidents in community pharmacies related to information technology, were about choosing the wrong drug. One third of incidents were associated with confusion of similar drug names and nearly half were associated with drug strength confusion [5]. Drug strength confusion can happen when two strengths of the same drug look alike, e.g. 3.75 mg and 0.375 mg pramipexole [6]. Drug name and strength confusion are serious issues as they are preventable errors with potential detrimental impact on clinical practice and patient safety [7,8].

**Fig 1:**
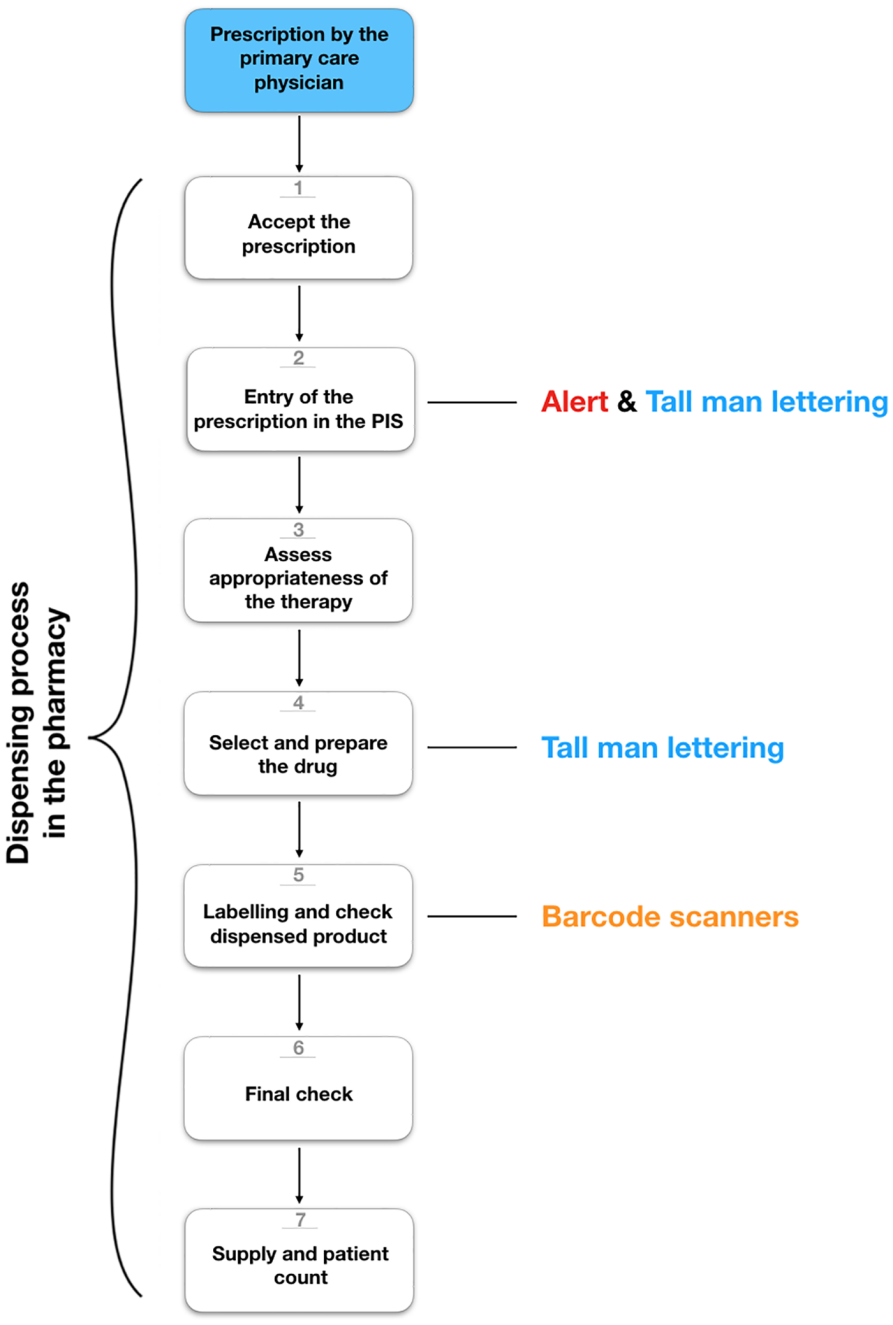
Different types of interventions to support the notification of potential drug name and drug strength confusion at different stages of the dispensing process in the pharmacies. DRP = drug-related problem, PIS = pharmacy information system. 1) Accept the prescription: verify the patient and the prescription; 2) Entry of the prescription in the PIS; 3) Asses appropriateness of the therapy: identify and resolve potential drug-related problems and review other aspects; 4) Select and prepare the drug; 5) Labeling and check dispensed product; 6) Final check by pharmacy technician or pharmacist; 7) Supply of the drug and patient counseling.

Several strategies can be implemented at different steps in the dispensing process to reduce the risk of medication errors because of drug confusion in pharmacies, such as the use of barcode scanners, Tall man lettering and alerts (Fig 1)[7,9–13]. Barcode scanners check whether the selected drug from the shelf is the same as the selected drug on the dispensing screen [7]. Tall Man lettering highlights the differences between look-alike drug names due to use of a combination of lower and upper case letters. The use of Tall man lettering is recommended in several countries [14–16], although evidence of a beneficial effect for Tall Man lettering is limited [10,17–19]. Alerts in the pharmacy information system (PIS) warn the pharmacist or pharmacy technicians for possible drug name or strength confusion at the time of entering the prescription in the PIS. This strategy has been implemented in Dutch community pharmacies [20].

A concern of this intervention is ‘alert fatigue’, a state caused by too many alerts resulting in alerts being completely ignored [21]. Since the number of potential alerts for drug name confusion is high [22], it is important to use the alert strategically [7,21,23]. Little is known about the effectiveness and how to use alerts for drug name and strength confusion in the PIS and whether they are seen as useful by pharmacy technicians and pharmacists.

### Objective

The aim of this study was to evaluate the experiences of pharmacists and pharmacy technicians with the alerts for drug name and strength confusion in the Netherlands.

## Methods

### Setting

#### Pharmacy information system (PIS)

The alert for drug name and drug strength confusion is implemented in one of the PIS in the Netherlands: Pharmacom^®^, by TSS PharmaPartners. This PIS is used in about 60% of the 1981 pharmacies in the Netherlands [24]. There are no alerts for drug name or strength confusion in any other PIS or in computerized prescribed order entry systems in the Netherlands.

#### Drugs with an alert for drug name and strength confusion

In 2015, the alert for drug name and strength confusion was revised. The selection of drugs with an alert was based on a list of most frequently reported and/or serious incidences in the period 2010–2015 at the Central Medication incidents Registration (CMR), and a qualitative evaluation in 9 pharmacies [20]. For a limited number of drugs (n = 8) an alert was implemented to prevent too many alerts, and reduce the risk of alert fatigue. Tall man lettering was used in three of the eight alerts for drug name confusion. For the drugs with an alert for strength confusion (5 alerts) only the highest strengths of the drugs received an alert to prevent alert fatigue. Morphine injections for which there was a risk of dispensing the wrong volume also received an alert (2 alerts)[20]. The drugs with an alert for name or strength confusion are listed in Table 1.

**Table 1:** Drugs with an alert for name and strength confusion [20].

**Drugs with an alert for drug name confusion**	
DexamethaSONe	DexamphetAMINE
Tobrex (tobramycin)	TobrADex (dexamethasone/tobramycin)
Fraxiparine^®^ (nadroparin)	Fraxodi^®^ (nadroparin)
estrADiol	Estriol
Modalim^®^ (ciprofibrate)	Modafinil (Modiodal^®^)
Haloperidol injection	Haloperidol decanoate injection (depot)
Povidone	Povidone iodine eye drops
Hydrochlorothiazide 25 mg	Hydroxyzine 25 mg
**Drugs with an alert for drug strength confusion**	
Pramipexole 0.375 mg	Pramipexole 3.75 mg
Levothyroxine 12.5 μg	Levothyroxine 125 μg
Haloperidol 0.5/1 mg	Haloperidol 5/10 mg
Morphine 10 mg/ml– 1 ml	Morphine 10 mg/ml– 10 ml
Morphine 20 mg/ml– 1 ml	Morphine 20 mg/ml– 5 ml
Desmopressin 2.5/10 μg/do	Desmopressin 150 μg/do
Oxazepam 5 mg	Oxazepam 50 mg

#### Alert in clinical decision support

If a prescription of one of the drugs listed in Table 2 was entered in the PIS, an alert was generated in case of a first dispensing. For example, in the case of prescription of dexamethasone, a pop-up window appeared with the text: ‘Caution for drug name confusion: DexamethaSONe or dexamphetAMINe?’. A single click abrogated the pop-up window and the dispensing process could be continued without interruption. The purpose of these pop-up windows was therefore to gain attention of the pharmacist or pharmacy technician. It was up to the person to act and check if the right drug or strength was selected.

**Table 2:** Pharmacist and technician’s experience with the alert for drug name and strength confusion.

Experience with the alerts.	Drug name confusion n, (%)	Strength confusion n, (%)
**Have you noticed the alert in your PIS?**	Yes: 230 (85.5)	Yes: 67 (25.5)
**How did you act when you noticed the alert?**		
- I checked if I had the right drug.	170 (74.6)	58 (89.2)
- I ignored the alert, because I knew I had the right drug.	35 (15.4)	3 (4.6)
- I asked a colleague to check if I had the right drug.	4 (1.8)	2 (3.1)
- I did not pay attention to the alert.	5 (2.2)	0 (0.0)
- Other.	14 (6.1)	2 (3.1)
**Has this alert prevented drug confusion in your pharmacy?**		
- Yes.	20 (8.8)	8 (12.3)
- No, because I had the right drug.	174 (76.3)	44 (67.7)
- Unknown.	34 (14.9)	13 (20.0)

Abbreviations: PIS = pharmacy information system.

### Study design

This was a cross-sectional survey of community pharmacists and pharmacy technicians in the Netherlands was performed in May 2017. A questionnaire was designed in the online survey software tool Survey Monkey (Survey Monkey, Palo Alto, CA, USA) and sent to all known e-mail addresses of potential Pharmacom users. For commercial and privacy reasons, e-mail addresses of Pharmacom users were not available. The collected e-mail addresses of potential users came from (1) subscribers to the Health Base Newsletter, that informs users about news concerning decision support and alerts in Pharmacom (n = 1234), and (2) Pharmacom users, that participated in a 2013 questionnaire study (n = 1056)[25]. The total number of invitations that were sent after removing duplicates (n = 2037) was higher than the estimated number of Pharmacom users (n = 1200), as the sample could contain more people within one pharmacy and also people not using Pharmacom. Those who did not want to participate were asked to give their reason for non-response. In total, three reminders were sent. Two reminders as an article in the Health Base Newsletter (1 and 4 weeks after the initial invitation), and one reminder was sent to all e-mail addresses that had not yet responded (2 weeks after initial invitation). The questionnaire was closed after 8 weeks.

This study was exempt from review by the institutional review board, since the study concerned healthcare professionals and did not involve any intervention. The data were collected anonymously and stored in accordance with privacy regulations.

### Questionnaire

Baseline characteristics were limited to the function within the pharmacy and the PIS that was used.

General questions for both name and strength confusion were: (1) have you noticed this alert, (2) if so, how did this influence the dispensing process, (3) did the alert prevent that a wrong drug was dispensed, (4) did the alert attract your attention, and—for drug name confusion–(5) how did you experience Tall man lettering, and (6) would you like Tall man lettering for all alerts concerning drug name confusion?

For the 15 alerts that were implemented in the PIS, respondents were asked if they preferred to receive an alert for this confusion and if they missed any drugs with potential name or strength confusion that should be added to the list.

Opinions about criteria to select a potential confusion as alert in the PIS were explored by eight statements that included statements about the potential to prevent harm versus the risk to introduce alert fatigue. For all questions, it was possible to add additional comments.

### Data analysis

All results from the online questionnaire were transferred into Microsoft Excel 2010 and IBM SPSS statistics 23.0. Frequencies and percentage, n (%), were computed. Differences between pharmacists and pharmacy technicians were calculated using Mann-Withney U test for variables on ordinal level, and Chi-Square Goodness of Fit Test for variables on nominal level. P-values of <0.05 were considered statistically significant. Comments on questions were analyzed and grouped if the comments were comparably. The proportion of respondents per pharmacy was calculated, assuming that for each pharmacy one respondent was reached with the collected e-mail addresses. Hence, the response rate per pharmacy was computed as the number of respondents divided by the number of Pharmacom pharmacies (n = 1200).

As the aim was evaluate the experience of the alerts for confusion of drug name and strength, respondents were asked to confirm if used the PIS Pharmacom. Those whom did not confirm, were excluded.

## Results

A total of 337 people responded to the invitation: 291 (86%) completed the questionnaire study and 46 (14%) completed a non-response questionnaire in which they gave their reasons why they did not participate in the study. The most common reasons were: 1) the respondents had not noticed that the alert had changed (n = 23), 2) they thought the alert was good as it is (n = 13) and 3) they had no time for answering the questionnaire (n = 10). Of the 291 participants in the questionnaire study, 22 (8%) were excluded because they did not use Pharmacom as PIS or because the used PIS was unknown.

Of the 269 included respondents, 193 (72%) were pharmacists and 76 (28%) were pharmacy technicians or pharmacy employees who had higher professional education at the bachelor level. The mean response rate per pharmacy was 22.3% (269/1200). As there were no noteworthy differences between pharmacists and technicians, the pooled results are presented.

### Experience with the alerts

Of the respondents, 86% had noticed the alert for drug name confusion in their PIS (Table 2). The alert for drug strength confusion was noticed by 26% of the respondents. Most of the respondents who noticed the alert checked if they had the right drug (75% for drug name confusion and 89% for strength confusion). Also 14 pharmacists (other) noticed the alert at the end of the day, at the final check and not during the dispensing process. The prevention of dispensing the wrong drug as result of the alert, had occurred in 9% of the pharmacies of the respondents who had noticed the alert for drug name confusion and 12% of the respondents who had noticed the alert for strength confusion.

The respondents indicated that they were satisfied with the drug name confusion alerts (Table 3). This varied between 97%, for dexamethasone and dexamfetamine, and 68% for hydrochlorothiazide and hydroxyzine. Most respondents (71%) did not feel the need for more alerts for drug name confusion. The most suggested drugs for an additional drug name confusion alert were lormetazepam and lorazepam (n = 9), and oxycodon immediate versus sustained release (n = 9). Also, the respondents indicated that they were satisfied with the alerts for strength confusion (Table 3). This varied between 93% for pramipexole 0.375 mg and 3.75 mg, and 64% for oxazepam 5 mg and 50 mg. Most respondents (81%) did not experience a need for more alerts for strength confusion. The most suggested drug for an additional drug strength confusion alert was atropine (eye drops 0.01% vs 0.1% vs 1%) (n = 6).

**Table 3:** Overview of the alerts for name and strength confusion and the proportion of respondents that is satisfied with the alert.

**Drugs with an alert for drug name confusion**		
**Would you like to receive an alert for** **drug name confusion** **for the following drugs?**		Yes; n, (%)
DexamethaSONe	DexamphetAMINE	254 (96.6)
Tobrex (tobramycin)	TobrADex (dexamethasone/tobramycin)	247 (94.3)
Fraxiparine^®^ (nadroparin)	Fraxodi^®^ (nadroparin)	243 (92.0)
estrADiol	Estriol	237 (90.1)
Modalim^®^ (ciprofibrate)	Modafinil (Modiodal^®^)	229 (87.4)
Haloperidol injection	Haloperidol decanoate injection (depot)	222 (84.7)
Povidone	Povidone iodine eye drops	206 (78.9)
Hydrochlorothiazide 25 mg	Hydroxyzine 25 mg	179 (68.1)
**Are there any other drugs whereby you also want to receive an alert for drug name confusion?**		77 (29.2)
**Drugs with an alert for drug strength confusion**		
**Would you like to receive an alert for** **strength and volume confusion** **for the following drugs?**		Yes; n, (%)
Pramipexole 0.375 mg	Pramipexole 3.75 mg	237 (93.3)
Levothyroxine 12.5 μg	Levothyroxine 125 μg	218 (85.8)
Haloperidol 0.5/1 mg	Haloperidol 5/10 mg	198 (78.0)
Morphine 10 mg/ml– 1 ml	Morphine 10 mg/ml– 10 ml	186 (73.2)
Morphine 20 mg/ml– 1 ml	Morphine 20 mg/ml– 5 ml	183 (72.0)
Desmopressin 2.5/10 μg/do	Desmopressin 150 μg/do	174 (68.5)
Oxazepam 5 mg	Oxazepam 50 mg	162 (63.8)
**Are there any other drugs whereby you also want to receive an alert for strength and volume confusion?**		49 (19.3)

### Notability of the alerts

Most of the respondents thought that the alerts were notable (89% for drug name confusion, 82% for strength confusion) (Table 4). To make the alert for drug name confusion more notable, respondents suggested to mark the difference between the drug names in color. Also, respondents commented that the pop-up window in the PIS was too small. Suggestions for making the alert for strength confusion more notable were to make the numbers bold or in color.

**Table 4:** Pharmacist and technician’s attitude towards the alert for drug name and strength confusion and the use of Tall man lettering.

Notability of the alerts.	Drug name confusion n, (%)	Strength confusion n, (%)
**Do you think the alert is striking?**	Yes: 236 (88.7)	Yes: 211 (81.5)
**Use of Tall man lettering**.		
**Does the use of Tall man lettering make it easier to note an alert?**	Yes: 249 (94.0)	
**I would like to see Tall man lettering**:		
- For all alerts concerning drug name confusion.	133 (50.2)	
- For drugs with the same memocode^a^.	71 (26.8)	
- Only for drugs with a high risk of confusion.	48 (18.1)	
- At no single alert for drug name confusion.	7 (2.6)	
- Other	6 (2.3)	

^a^ Memocode is used to find the right drug easier and quicker in the PIS. A memocode in Pharmacom consist of the first five letters of the drug followed by two or three numbers or letters corresponding to the administration form or the strength (e.g. paracetamol tablet 500mg = PARAC 500).

Tall man lettering was experienced as an improvement in the alerts for drug name confusion by 94% of the respondents (Table 4), because this made the differences between the names more distinctive and the alert more notable. Half of the respondents preferred Tall man lettering in every alert for drug name confusions. Respondents who thought that Tall man lettering was not improving the alert commented that the plain letters forced you to read better.

### Criteria for an alert for drug confusion

Most of the respondents preferred to receive an alert for drug name or strength confusion (91%). Both the seriousness of the consequences (90%) as the frequency of the reported incidents (82%) were factors that contributed to the need to prefer an alert. Respondents did not necessarily want to receive an alert for every incident. Of the respondents, 45% found it sufficient if the alert would only be received at the first dispensing. An important comment on the questionnaire was that pharmacies indicated that the error is, most of the time, already made during prescription by the primary care physician. Therefore, they thought it was important that the alert would also be generated in the system of the prescribers.

### Alert fatigue

About half of the respondents (52%) did not favor removing an alert for a specific drug if there were no incidents due to drug confusion in the last year. The reason for this was that the absence of incidences could have been due to the alert, and removal of the alert could reinstate drug confusion. Moreover, respondents thought that alerts of confusions that caused serious incidents should stay.

According to the additional comments (n = 35) that were given, ‘alert fatigue’ was an important issue and this should be prevented. In the open answers respondents insisted they preferred not (too) many alerts, as this increased the risk of alerts being ignored. However, there was no maximum number of alerts for drug name or strength confusion because an alert for each drug with a high risk for confusion and/or with serious consequences was desired. Respondents indicated that there could be various reasons not to alert a drug name confusion that resulted in a serious case (72%). The reason given most frequently was ‘an odd drug confusion’, such as ‘bisoprolol—lisinopril’(36%).

## Discussion

Pharmacies were satisfied with the alerts, and experienced that the alerts for drug name and strength confusion contributed to the prevention of medication errors. This suggests that this strategy of using alerts is useful in addition to other strategies, such as barcode scanning and checking of the dispensing process by a colleague [7]. To minimize risks in complex processes such as drug dispensing, a combination of safety measures was found to be most effective. Since drug confusion is an important contributor to the drug dispensing error rate, implementation of alerts for drug name and strength confusion could significantly reduce this error rate [3,8,26].

For three alerts, less than 70% of the respondents indicated they preferred to receive that alert. A possible reason is the fear for ‘alert fatigue’, because these drugs (oxazepam and hydrochlorothiazide) are regularly prescribed in the Netherlands. This indicates that an alert for drug name and strength confusion is valuable, but that not every drug name or strength confusion needs an alert. Therefore, it is necessary to determine the best strategy for the use of these alerts. Selection criteria should be established about the implementation of new alerts for drug confusion and removal of unnecessary alerts. Cases of name and strength confusion and experiences of pharmacies and prescribers with alerts, including alert fatigue, should be evaluated regularly (annually) to decide about the implementation of new alerts and removal of alerts.

Pharmacies preferred to receive alerts for drugs where confusions occur frequently or are associated with serious consequences. In the case of a non-serious incident or an odd confusion an alert is not desired. However, the definition of an ‘odd confusion’, ‘frequent occurrence’ and ‘serious consequences’ is unclear.

The respondents experienced the notability of the alerts as sufficient and that the use of Tall man lettering improved drug name confusion alerts. This is in accordance with other studies that suggests that Tall man lettering makes similar names easier to distinguish [8,27]. Given the positive experience with Tall man lettering, this should be used in the alerts for drug name confusion. In the text of the alerts the specific drug names and the strengths that could be confused were named. It remained unclear whether this information was useful. Further research should investigate how the alerts should be presented in order to have the highest chance of being noticed (Tall man lettering, larger pop-up window etc.).

This is the first study that evaluated alerts for drug name and strength confusion in the PIS. In this study alerts for drug name and drug strength confusion were evaluated that were implemented in one of the PIS in the Netherlands, used by 60% of the pharmacies. A total of 269 people working in community pharmacy, both pharmacists and pharmacy technicians, participated in this survey. However, this study has some limitations. Although this study had a mean proportion of 22.3% respondents per pharmacy, the response rate is low compared to the number of questionnaires that were sent, and response rates of from around 30% found in other studies using web-based questionnaires [28–30]. Several reasons can explain this: the email list was incomplete, the questionnaire was mailed to a general email address without addressing the questionnaire to a responsible employee, and pharmacies might have unsubscribed to the newsletter. Another explanation might be that the alerts met the expectations, as the results showed that the respondents were satisfied. Furthermore, only pharmacies that had implemented the alert for drug confusion were investigated, and no other pharmacies or prescribers. Hence, the results might not be representative for all pharmacies. Yet, given the limitations, this study is a legitimate approach to expand the knowledge on preventing drug confusion.

Further research should determine the interception rate (measure of the potential benefit of the intervention) of alerts for drug name and strength confusion in the PIS is determined. Such a study should demonstrate the actual effectiveness of the alerts on the dispensing error rate. Also, future research should focus on prescribers: an alert for drug name and strength confusion in the computerized prescribed order entry system will probably have additional value to the current alert in the PIS.

## Conclusion

Pharmacists and pharmacy technicians notice alerts for drug name and drug strength confusion in their PIS and experience that these alerts contribute to the prevention of medication errors. Alert fatigue is an important issue: pharmacies prefer to have an alert in case of confusions that have been reported frequently, or were associated with serious safety incidents. It was considered important not to include all possible confusions as a new alert.
